# Genotyping and inflated type I error rate in genome-wide association case/control studies

**DOI:** 10.1186/1471-2105-10-68

**Published:** 2009-02-23

**Authors:** Joshua N Sampson, Hongyu Zhao

**Affiliations:** 1 Department of Epidemiology and Public Health, Yale University School of Medicine, New haven, CT, USA

## Abstract

**Background:**

One common goal of a case/control genome wide association study (GWAS) is to find SNPs associated with a disease. Traditionally, the first step in such studies is to assign a genotype to each SNP in each subject, based on a statistic summarizing fluorescence measurements. When the distributions of the summary statistics are not well separated by genotype, the act of genotype assignment can lead to more potential problems than acknowledged by the literature.

**Results:**

Specifically, we show that the proportions of each called genotype need not equal the true proportions in the population, even as the number of subjects grows infinitely large. The called genotypes for two subjects need not be independent, even when their true genotypes are independent. Consequently, p-values from tests of association can be anti-conservative, even when the distributions of the summary statistic for the cases and controls are identical. To address these problems, we propose two new tests designed to reduce the inflation in the type I error rate caused by these problems. The first algorithm, logiCALL, measures call quality by fully exploring the likelihood profile of intensity measurements, and the second algorithm avoids genotyping by using a likelihood ratio statistic.

**Conclusion:**

Genotyping can introduce avoidable false positives in GWAS.

## Background

One common goal of a genome wide association (GWAS) study is to search the entire genome for single nucleotide polymorphisms (SNPs) and copy number variations (CNVs) associated with a disease or some other phenotype. In this article, we focus our analysis on SNPs. The two possible alleles at a SNP are arbitrarily labeled A and B, and association is often tested by measuring and comparing the frequencies of the genotypes AA, AB, and BB, in case and control groups. As technology currently allows close to one million SNPs to be examined simultaneously, there is a need for fast, automated methods to test for association. As only a small minority of SNPs are expected to be associated with the disease, even a modest false positive rate could bury true associations beneath those occurring by chance.

Using Affymetrix 500 k GeneChips as an example, each of the 500,000+ SNPs is represented by a series of probes on a pair of arrays. Each probe is an oligonucleotide designed to bind to either the A or B allele. A subject's fluorescently labeled DNA is allowed to hybridize with these probes, and then a spectrometer measures the relative fluorescent levels between the A and B probes. Each genotyping algorithm (see *Methods*) summarizes the fluorescence information, or the likelihood a subject has allele A at that SNP, by its own statistic. In any population, these statistics usually cluster into three groups, corresponding to the three genotypes. Studies have noted that 1) The mean and variance of these clusters, or the shape of their distribution in general, varies by SNP and 2) For a single SNP, because of differences in processing or duration of storage, the shape of the statistic's distribution can differ between the case and control groups [1-3].

The current test of association requires *calling*, or assigning a genotype, to each SNP for each study subject and then comparing the called genotypes between the case and control groups. The majority of SNPs are easy to call and any of the available methods will call them correctly. Unfortunately, there is a difficult minority that cannot be easily clustered into three distinct groups. Because there can be as many as 500,000 SNPs, this minority can greatly inflate the type I error rate and cause the large, characteristic, deviations from the x = y line in the qq-plots of test statistics. Most studies assume these consequences are the unavoidable results of population substructure and poor data. In this paper, we dispel the myth that these are the sole issues. In fact, the inflated type I error rate and general misbehavior of the test statistic may also result from the act of genotype assignment and a poor choice of statistical methodology.

The goal of this manuscript is two-fold. First, our primary goal is to show that genotyping overlapping clusters can lead to potential problems that we have yet to see fully acknowledged in the literature. The proportions of each called genotype need not equal their true proportions in the population, *even as the number of subjects grows infinitely large*. As we compare genotype calls, p-values from tests of association will be anti-conservative when the distribution of the summary statistic differs between cases and controls. Moreover, the called genotypes for two subjects need not be independent, *even when their true genotypes are independent*. Therefore, p-values from tests of association can be anti-conservative, *even when the distributions of the summary statistic for the cases and controls are identical*, a fact we believe has yet to be fully demonstrated. Although previous studies have examined the effects of genotyping error on tests of association [4-6], studies has neither fully explored the effects caused by case/control differences in distributions nor dependence of error. Second, we discuss two new tests that can circumvent these potential problems. One test compares calls made from a genotyping algorithm designed to minimize the type I error. The second test compares the fluorescence distributions, instead of the called genotypes. We start the *Methods section *by discussing currently available methods for genotyping SNPs and testing for association. Concurrently, we introduce logiCALL, our new genotyping algorithm, and the likelihood ratio-based test of association. In the *Results and Discussion section*, we start by showing that the proportion of genotypes called AA, AB, and BB need not converge to the true population proportions. Then we discuss how the called genotypes can be dependent. We conclude the section by comparing the proposed tests of association with the current standards through both simulation studies and real data analysis. Then a short *Conclusions section *summarizes the key points.

## Methods

### Calling Genotypes

There is currently a wide variety of programs available for genotyping SNPs. The most popular supporting Affymetrix are RLMM [7], BRLMM [8], CRLMM [9], CHIAMO [10], SNiPer-HD [11], and MAMS [12]. The most popular program for Illumina is their own proprietary software, BeadStudio, but other methods have been recently suggested by Moorhead et. al. [3], Teo et. al. [13], and Dunning et. al. [14] (Table 1). To introduce these methods, we start by defining notation. Let there be *n *subjects. Let *G*_*ij *_∈ {*AA*, *AB*, *BB*} be the true genotype of SNP *j *in subject *i*, 1 ≤ *i *≤ *n*. For Affymetrix chips, assume there are *n*_*p *_probe quartets representing each SNP on an array, and let ${\vec{I}}^{ijk}\equiv \{I_{PM_{A}}^{ijk},I_{MM_{A}}^{ijk},I_{PM_{B}}^{ijk},I_{MM_{B}}^{ijk}\}$ be the normalized probe intensities for subject *i*, SNP *j*, and probe *k*. Here, the subscripts *PM*_*A *_and *MM*_*A *_signify the perfect match and mismatch probes for allele A. *PM*_*B *_and *MM*_*B *_are similarly defined for allele B. The log transformed intensities will be ${\vec{Y}}^{ijk}=log_{2}({\vec{I}}^{ijk})$. To maintain notational consistency, for Illumina chips, we denote the BeadStudio intensity values of the two SNP alleles by $\left\{I_{PM_{A}}^{ij},I_{PM_{B}}^{ij}\right\}$. As we will only discuss a single SNP for the majority of the paper, we will omit the superscript "*j*" from all future notation.

**Table 1:** Programs available for genotyping SNPs.

Name	Summary Statistic	MM^1^	Data^2^	Data^3^	Notes
RLMM	{Θ_*A*_, Θ_*B*_}	No	T	M	
BRLMM	$\frac{1}{asinh(4)}asinh\left(\frac{4(I_{A}-I_{B})}{I_{A}-I_{B}}\right)$	No	E-U	M	Assumes genotypes in "training" data are known. "Training" data only uses high quality SNPs. Incorporates info from other SNPs as a *B*ayesian Prior.
CRLMM	{Θ_*A*+ _- Θ_*B*+_, Θ_*A*- _- Θ_*B*-_}	No	T	L	*C*orrects for the effect of total intensity level and probe length on {*S*_*ij*_} through more complex method, and allows corrections to vary by array.
CHIAMO	$\left\{\frac{1}{np}\sum_{k=1}^{n_{p}}Y_{Ak},\frac{1}{np}\sum_{k=1}^{n_{p}}Y_{Ak}\right\}$	Yes	E-L, T*	W	CHIAMO is a Bayesian hierarchical mixture model and is greatly simplified by this brief summary
SNiPer-HD	$\begin{array}{l} \{R_{1},...,R_{n_{p}}\},\text{ where}\\ R_{k}=(I_{PM_{A}}^{ijk})/(I_{PM_{A}}^{ijk}+I_{PM_{B}}^{ijk}) \end{array}$	No	E-U	W	Assumes genotypes in "training" data are unknown and requires the EM algorithm. "Training" data should only use high quality SNPs.
Moorhead	$\frac{1}{sinh(2)}sinh\left(\frac{I_{PM_{A}}+I_{PM_{B}}}{I_{PM_{B}}}\right)$	N/A	E-U	W	Originally for MIP, but applicable to Affymetrix. Plagnol demonstrated how to link genotype probabilities between cases and controls.
logiCALL	$\left\{\frac{1}{sinh(2)}sinh\left(\frac{I_{PM_{A}}+I_{PM_{B}}}{I_{PM_{B}}}\right),I_{PM_{A}}+I_{PM_{B}}\right\}$	No	E-L	W-F	Designed to lower false positive rate and assigns calls based on cumulative distribution, not density functions.

^1^Indicates use of mismatched probes

^2^Parameters were estimated by *E*xperimental or *T*raining data. For experimental data, under the null, cases and control genotype proportions could be *L*inked or *U*nlinked. * indicates optional.

^3^Distance can be *M*ahalanobis, *W *eighted Likelihood, or unweighted *L*ikelihood

With the exception of dynamic modeling (DM) [15], all calling algorithms share the same general form, and we exploit this form to summarize their key features. The process of transforming raw signal into genotypes can be divided into four steps: 1) Normalize the intensity values; 2) Describe the normalized intensity values by a single, possibly multivariate, summary statistic; 3) Estimate the mean and variance of the summary statistic for the three possible genotypes, AA, AB, and BB; and 4) Compare the value of the statistic from a subject to the group parameters found in the third step to make the call. The universal first step, normalizing the intensity values, is tangential to our discussion here. The second step is to choose a statistic, *S*_*i *_that Summarizes the intensities. For example, RLMM models the probe intensities as $Y_{PM_{A}}^{ik}=\zeta_{A}^{i}+\beta_{A}^{k}+\varepsilon_{A}^{ik}$ and $Y_{PM_{B}}^{ik}=\zeta_{B}^{i}+\beta_{B}^{k}+\varepsilon_{B}^{ik}$. Then, $S_{i}\equiv \{\zeta_{A}^{i},\zeta_{B}^{i}\}$. In the updated version, CRLMM models sense(+) and antisense(-) probes separately, resulting in a 4D statistic, $S_{i}\equiv \{\zeta_{A+}^{i},\zeta_{A-}^{i},\zeta_{B+}^{i},\zeta_{B-}^{i}\}$ Each method assumes that the distribution, *φ*^*M*^(*S*_*i*_|*θ*^*q*^), of their statistic in a given population *q *is a Mixture of multivariate normal distributions where *θ*, to be defined below, are the parameters characterizing the distribution in population *q*. When it is clear we are discussing only a single population, we will omit the superscript *q*. Although problems can arise when the distribution is not a true mixture of normals, those complications are beyond the scope of this paper [16]. For completeness, we point out that a minority of programs, including CRLMM, allow these parameters to vary within a group (e.g. to be subject/array specific). Ignoring that some methods allow for an additional null distribution, the general form is

(1) $$\begin{array}{ll} \phi^{M}(S_{i}|\theta )= & p_{AA}\phi (S_{i}|{\vec{\mu }}_{AA},\Sigma_{AA})+\\ & p_{AB}\phi (S_{i}|{\vec{\mu }}_{AB},\Sigma_{AB})+\\ & p_{BB}\phi (S_{i}|{\vec{\mu }}_{BB},\Sigma_{BB}) \end{array}$$

where *ψ*(·) is the multivariate normal density. The three mean vectors, $\mu \equiv \{{\vec{\mu }}_{AA},{\vec{\mu }}_{AB},{\vec{\mu }}_{BB}\}$, variance matrices, Σ ≡ {Σ_*AA*_, Σ_*AB*_, Σ_*BB*_}, and probabilities, *p *≡ {*p*_*AA*_, *p*_*AB*_, *p*_*BB*_}, correspond to the three possible genotypes, AA, AB, and BB. Define $\theta \equiv \{\vec{\mu },\Sigma ,\vec{p}\}$ and later, we let Φ(·) and Φ^*M*^(·) be the cumulative distribution for a normal variable and a mixture of normals. The third step is to estimate *θ*. Some algorithms, such as RLMM, use a training data set, where the genotypes are known. Other algorithms, such as SNiPer-HD, use no training data, and find the best estimates that describe their experimental values. The fourth step is to assign a genotype, ${\hat{G}}_{ij}$, to SNP *j *in subject *i*. Often, for a given value of *S*_*i*_, the assigned genotype maximizes a similarity function: ${\hat{G}}_{i}$ = *argmax*_*g *_*D*(*g, S*_*i*_|*θ*). The similarity function is usually a modified version of one of the following three quantities: Mahalanobis distance: $-(S_{i}-{\vec{\mu }}_{g})\Sigma_{g}^{-1}{\left(S_{i}-{\vec{\mu }}_{g}\right)}^{t}$, Unweighted Likelihood: $\phi (S_{i}|{\vec{\mu }}_{g},\Sigma_{g})$, or the Weighted Likelihood: *p*_*g*_$\phi (S_{i}|{\vec{\mu }}_{g},\Sigma_{g})$. The similarity function is also modified to ensure monotonicity of assignment. When we let *S*_*i *_= {*M*_*i*_, *A*_*i*_}, we force *D*(*AB, S*_*i*_|*θ*) = *D*(*BB, S*_*i*_|*θ*) = 0 if *M*_*i *_≤ *μ*_*AA*_, *D*(*BB, S*_*i*_|*θ*) = 0 if *μ*_*AA *_≤ *M*_*i *_≤ *μ*_*AB*_, *D*(*AA, S*_*i*_|*θ*) = 0 if *μ*_*AB *_≤ *M*_*i *_≤ · *μ*_*BB*_, and *D*(*AB, S*_*i*_|*θ*) = *D*(*AA, S*_*i*_|*θ*) = 0 if *M*_*i *_≥ *μ*_*BB*_, where *μ*_*g*_, in this case, is the mean of *M*_*i *_when *G*_*i *_= *g*. This modification is standard in calling algorithms. As we do not know the true value of the parameters in experiments, we replace *D*(*g*, *S*_*i*_|*θ*) by *D*(*g*, *S*_*i*_|$\hat{\theta }$). A subject's SNP may not be called, or assigned a missing value, if the difference or ratio between *D*(${\hat{G}}_{i}$, *S*_*i*_|$\hat{\theta }$) and *D*(*g*_2*i*_, *S*_*i*_|$\hat{\theta }$) is not large enough, where *g*_2*i*_is the genotype with the second largest value of *D*(*g*, *S*_*i*_|$\hat{\theta }$). A SNP may be omitted from further study if too many values were set to missing. Table 1 describes the details of the four steps for popular methods. For many purposes or to understand the details of the method, especially in handling rare alleles, this table will seem an oversimplification. For our purposes here, it highlights the features of interest.

### Tests of Association

The current tests of association start by calling genotypes for a given SNP *j *in a group of subjects with the disease and in a group of controls. They then compare the resulting proportions, ${\hat{P}}^{A}\equiv \{{\hat{P}}_{AA}^{A},{\hat{P}}_{AB}^{A},{\hat{P}}_{BB}^{A}\}$ and ${\hat{P}}^{U}\equiv \{{\hat{P}}_{AA}^{U},{\hat{P}}_{AB}^{U},{\hat{P}}_{BB}^{U}\}$, from these Affected and Unaffected groups using either a Cochran-Armitage test or logistic regression. Here ${\hat{p}}_{g}^{q}\equiv \frac{1}{nq}\sum_{i:Q_{i=q}}1({\hat{G}}_{i}=g)$, where the indicator function is defined by 1(x) = 1 if x is true, 0 otherwise, *Q*_*i *_∈ {*A*, *U*} is the disease status for individual *i*, and *n*_*q *_is the number of subjects with disease status *q*. In this manuscript, any 'p-value' from a genotype-based association test will be calculated using ANOVA on the logistic regression model with *Q*_*i *_and genotype (unordered grouping) as the dependent and independent variables.

Standard tests tend to err anti-conservatively as we will discuss below. We will propose four alterations that can reduce type I error rate, with only a minimal decrease in power. These are the four differences that separate logiCALL from standard methods. The first is based on the observation, which is discussed later, that the likelihood profile of *φ*^*M*^(*S*_*i*_|*θ*) will have multiple local maxima near the overall maximum. When estimating *θ*, the EM algorithm converges to multiple solutions. For many of those solutions, the resulting parameter set, ${\hat{\theta }}_{lm}$, satisfies $\prod_{i}\phi^{M}(S_{i}|\hat{\theta })\leq \prod_{i}\phi^{M}(S_{i}|{\hat{\theta }}_{lm})+\tau n$. For each parameter set satisfying this inequality, we will make a new group of genotype assignments, $\left\{{\hat{G}}_{i}^{lm}\right\}$. If more than 10% of such assignments disagree with ${\hat{G}}_{i}$ (*τ *= 0.06), we label that subject's call as questionable. We also continue the practice of marking calls with small values of *D*(${\hat{G}}_{i}$, *S*_*i*_|$\hat{\theta }$)/*D*(*g*_2*i*_, *S*_*i*_|$\hat{\theta }$) as questionable. The second alteration is that we do not discard questionable calls, an act which can create false positives. Instead, we assign questionable *S*_*i *_so the proportions of genotypes in the cases and controls are as similar as possible, which is defined as minimizing $\sum_{g}\left|{\hat{p}}_{g}^{U}-{\hat{p}}_{g}^{A}\right|$, with the restriction that the final call for subject i must be either ${\hat{G}}_{i}$ or *g*_2*i*_. Here, we let *g*_2*i *_be the genotype which is either the runner-up in terms of distance or the most common genotype among the dissenting calls, depending on why the genotype was labeled as questionable. The third alteration, which is already incorporated into other programs is to perform the EM algorithm under the null hypothesis that the genotype proportions in the two populations are identical [3]. The fourth is the use of a weighted Mahalonobis distance, which is defined later. Given these changes, logiCALL then compares the estimated genotype proportions in cases and controls using logistic regression. Note that none of the changes affect calls for the vast majority of SNPs.

We also introduce a completely new method for testing association based on a likelihood ratio statistic. For our method, steps 1 and 2, normalization and choice of summary statistic, can mimic any of the previously described methods. As our real data to be analyzed was collected on Illumina chips, we choose the statistic from Moorhead, et al. [3], $S=\frac{1}{sinh(2)}sinh(\frac{I_{PM_{A}}+I_{PM_{B}}}{I_{PM_{B}}})$ for exposition. We then assume that *S*_*i *_follows the mixture model described by equation (1), but allow the parameters to differ by disease status:

(2) $$\phi_{AU}^{M}(S_{i}|\theta )\equiv \{\begin{array}{c} \phi^{M}(S_{i}|\theta^{A}),Q_{i}=A\\ \phi^{M}(S_{i}|\theta^{U}),Q_{i}=U \end{array}$$

and *θ *= {*θ*^*A*^, *θ*^*U*^}, where

$$\begin{array}{l} \theta^{A}=\left\{p_{AA}^{A},\mu_{AA}^{A},\sigma_{AA}^{2A},p_{AB}^{A},\mu_{AB}^{A},\sigma_{AB}^{2A},p_{BB}^{A},\mu_{BB}^{A},\sigma_{BB}^{2A}\right\}\\ \theta^{U}=\left\{p_{AA}^{U},\mu_{AA}^{U},\sigma_{AA}^{2U},p_{AB}^{U},\mu_{AB}^{U},\sigma_{AB}^{2U},p_{BB}^{U},\mu_{BB}^{U},\sigma_{BB}^{2U}\right\} \end{array}$$

Although *θ *contains 18 parameters, it has only 16 degrees of freedom (df) because $p_{AA}^{q}+p_{AB}^{q}+p_{BB}^{q}=1$ for *q *∈ {*A, U*}. Our new test will reject the null hypothesis of no association, when *LR*($\vec{S}$), the likelihood ratio, is large, where

(3) $$LR(\vec{S})=(\frac{max_{\hat{\theta }\in \Omega }\prod \phi_{AU}^{M}(S_{i}|\hat{\theta })}{max_{{\hat{\theta }}_{R\in \Omega R}}\prod \phi_{AU}^{M}(S_{i}|{\hat{\theta }}_{R})})$$

Clearly, the restricted parameter space, $\left\{\Omega_{R}:p_{AA}^{A},p_{AA}^{U},p_{AB}^{A}=p_{AB}^{U},p_{BB}^{A}=p_{BB}^{U}\right\}$ is a subset of the unrestricted parameter space, Ω. In an ideal scenario, the distribution of 2log(LR) would converge to a chi-squared distribution, $F_{\chi_{2}^{2}}$, with 2 degrees of freedom. Therefore, the 'p-value' from a likelihood ratio-based test will be calculated as 1 - $F_{\chi_{2}^{2}}$(2*log*(*LR*)).

### Data Source

To demonstrate the problems of genotyping and compare the genotype- and likelihood ratio-based tests of association, we use three types of data. First, for discussion, we may assume a hypothetical study measuring a one dimensional summary statistic, *S*_*i*_, for a SNP *j *with only two possible genotypes, *G*_*i *_∈ {0, 1}. Furthermore, to show problems can exist even under the best conditions, where model and truth coincide, we assume that *S*_*i *_follows a normal distribution given *Q*_*i *_and *G*_*ij*_, and that the full distribution can be described by $\phi^{M}(S_{i}|\theta )=p_{0}\phi (S_{i}|\mu_{0},\sigma_{0}^{2})+p_{1}\phi (S_{i}|\mu_{1},\sigma_{1}^{2})$.

We compare our two new tests of association to a standard method using simulated data. The standard method mimics the general Bead-Studio approach by a) fitting parameters with the EM algorithm; b) calling genotypes based on the Mahalanobis Distance; c) removing all calls where *D*(${\hat{G}}_{i}$, *S*_*i*_|$\hat{\theta }$)/*D*(*g*_2_, *S*_*i*_|$\hat{\theta }$) > 0.5; and d) comparing the two sets of resulting estimates, $\left\{{\hat{P}}_{AA}^{A},{\hat{P}}_{AB}^{A},{\hat{P}}_{BB}^{A}\right\}$ and $\left\{{\hat{P}}_{AA}^{U},{\hat{P}}_{AB}^{U},{\hat{P}}_{BB}^{U}\right\}$. We generated 10 simulated datasets, containing 1000 subjects (500 cases, 500 controls) and 303,100 SNPs for each of 18 scenarios. For each gene *j *and each subject *i*, we generated a 2D summary statistic (*M*_*ji*_, *A*_*ji*_). The distribution of *M_*ji *_*depended on genotype. If *G*_*ji *_= *AA*, then *M*_*ji *_~ 2*X *- 1, and if *G*_*ji *_= *BB*, then *M*_*ji *_~1 - 2*X*, where *X *~ *beta*(*α *= 3, *β *= 30). If *G*_*ji *_= *AB*, then *M*_*ji *_also followed a beta distribution, but the parameters varied by SNP, disease status, and scenario. For all SNPs and all subjects, *A_*ji *_*~ *N *(10, 1.5). We generated three types of SNPs, background, shifted, and influential. First, 300,000 background SNPs were generated and included in all 10 × 18 = 180 data sets. For each SNP, a single minor allele frequency was generated from a uniform(0.2, 0.4) distribution and genotype probabilities $\left({\vec{p}}^{U}={\vec{p}}^{A}\right)$ were generated assuming Hardy-Weinberg Equilibrium. Here, *E *[*M*_*ji*_|*G*_*ji *_= *AB*] = 0 and was independent of disease status. These SNPs, which formed three distinct clusters, can be easily identified and represent a well-behaved group. For 3,000 shifted SNPs, MAF ~*uniform*(0.2, 0.4) and genotype probabilities $\left({\vec{p}}^{U}={\vec{p}}^{A}\right)$ were generated assuming Hardy-Weinberg Equilibrium. Here, *E *[*M*_*ji*_|*G*_*ji *_= *AB*] ∈ {-0.739 + 0.2, - 0.739 + 0.*3 *- 0.739 + 0.5}, where we note *E *[*M*_*ji*_|*G*_*ji *_= *AA*] = -0.739 and *E *[*M*_*ji*_|*G*_*ji *_= *AB*, *Q*_*i *_= *A*] - *E *[*M*_*ji*_|*G*_*ji *_= *AB*, *Q*_*i *_= *U*] ∈ {0, 0.2}. This group represents difficult to call SNPs. For 100 influential SNPs, MAF ~*uniform*(0.2, 0.4) and genotype probabilities $\left({\vec{p}}^{U}\neq {\vec{p}}^{A}\right)$ were chosen so that, under a disease prevalence of 0.01 and a model of additive effects, the genotype relative risk for subjects homogeneous for the minor allele, *P*(*Q*_*i *_= *A*|*BB*)/*P *(*Q*_*i *_= *A*|*AA*) ∈ {1.5, 2.0, 2.5}. Combing the degree of shift for poor quality SNPs and the effect size of truly associated SNPs, we have a total of 18 scenarios used in our simulation.

The next set of data is from a recent GWAS of Inflammatory Bowel Disease (IBD) that compared 983 subjects with IBD to 1004 subjects without the disease. Using Illumina microarrays, 308,330 SNPs on the autosomal chromosomes were tested. Jewish and non-Jewish cohorts, approximately equal in size, were analyzed separately, a practice continued here. Details have been previously published [17,18]. Because the overwhelming majority of the SNPs are easy to genotype, as any of the summary statistics neatly divide into three clusters, we chose a 3137 *difficult *SNP subset where at least two clusters overlap (definition below). Because association was tested separately in Jewish and non-Jewish cohorts, a total of 3137 × 2 = 6274 tests were possible. To demonstrate called genotype dependency, we bootstrapped 40 samples, ignoring case/control status, of 500 subjects for each SNP. A sample would be discarded, and replaced by another random selection, if one or both of the groups were lacking an AA (*S*_*i*_<-0.7) or a BB (*S*_*i *_> 0.7) genotype.

We defined *difficult *SNPs as follows. For SNP *j*, we first estimated the density, $\hat{f}$(*M*_*j*._) nonparametrically using the R function 'density(adjust = 0.3)'. In theory, $\hat{f}$(*M*_*j*._) is a mixture of three normal distributions, corresponding to the three genotypes. If the SNP is well-behaved, then the three underlying densities will not overlap, and the empirical density $\hat{f}$(*M*_*j*._) will attain minima near 0 in the valleys between *μ*_*jAA *_and *μ*_*jAB *_and between *μ*_*jAB *_and *μ*_*jBB*_. If either of these minima exceeded 0.2, then at least two of underlying densities overlapped, and that SNP was defined as *difficult*. To speed the process, we found that approximating the center of the peaks (i.e. *μ*_*jAA*_, *μ*_*jAB*_, and *μ*_*jBB*_) by the median values of *M*_*j*. _in the three windows, {*M*_*j*. _≤ -0.6; -0.3 ≤ *M*_*j*. _≤ 0.3, *M*_*j*. _≥ 0.5}, worked well.

## Results and discussion

### Two Genotype Example: Parameters

We choose to use the hypothetical, two-genotype, study, to highlight that the estimated parameters can be inconsistent even in the simplest scenario, where the summary statistic is distributed normally and our fitted model is correct. When dealing with only a single population, we define the parameter *p*_0 _(*p*_1_) to be the probability that a subject's *true *genotype is 0 (1).

(4) *p*_0 _≡ *P *(*G*_*i *_= 0) and *p*_1 _≡ *P*(*G*_*i *_= 1)

We define the parameter $p_{0}^{n*}(p_{1}^{n*})$ to be the probability that a subject's *called *genotype is 0 (1).

(5) $$p_{0}^{n*}\equiv P({\hat{G}}_{i}=0|n,\theta )\text{ and }p_{1}^{n*}\equiv P({\hat{G}}_{i}=1|n,\theta )$$

Probabilities of called genotypes implicitly depend on the number of subjects in the sample. We then define the parameter c* to be the point which is equidistant to the two genotype groups when *θ *is known.

Therefore, *μ*_0 _≤ c* ≤ *μ*_1 _is defined to be a solution to equation 6

(6) *D*(*G*_*i *_= 0, *S*_*i *_= c*|*θ*) = *D*(*G*_*i *_= 1, *S*_*i *_= c*|*θ*)

For the remainder of the paper, we shall assume such a c* exists. This assumption is safe in practice as genes with extremely rare minor alleles are discarded. When D is the Mahalanobis distance, c* is a solution to

(7) $$\frac{{\left(c*-\mu_{0}\right)}^{2}}{\sigma_{0}^{2}}=\frac{{\left(c*-\mu_{1}\right)}^{2}}{\sigma_{1}^{2}}$$

We define the final parameter, $p_{0}^{*}(p_{1}^{*})$, to be the probability that a subject's *S*_*i *_value is less than c* given their true genotype is 0 (1).

(8) $$\begin{array}{l} p_{0}^{*}\equiv P(S_{i}<c*|G_{i}=0,\theta )\\ p_{1}^{*}\equiv P(S_{i}<c*|G_{i}=1,\theta ) \end{array}$$

Our first goal in this *Results section *is to show that

(9) $$p_{0}^{*}\neq p_{0}$$

may be true when two clusters, {*S*_*i*_:*G*_*i *_= 0} and {*S*_*i*_:*G*_*i *_= 1} overlap. We will refer to $p_{0}^{*}$ - *p*_0 _as asymptotic bias, or bias, and we note that it depends on D, *p*_0_, and the magnitude of the overlap. Here, we also define C, our estimate for c*, to be the solution to the equation

(10) $$D(G_{i}=0,S_{i}=C|\hat{\theta })=D(G_{i}=1,S_{i}=C|\hat{\theta })$$

such that ${\hat{\mu }}_{0}\leq C\leq {\hat{\mu }}_{1}$. If no such C exists, to be consistent with monotonicity of assignment, we let $C={\hat{\mu }}_{g}$ where *D*(*G*_*i *_= *g*, *S*_*i *_= *C*$\hat{\theta }$) is the smaller of the two measures when ${\hat{\mu }}_{0}\leq S_{i}\leq {\hat{\mu }}_{1}$. The variable C is the *cut-point *or threshold value of *S *which separates 0 and 1 calls. Therefore, by monotonicity of assignment, *S*_*i *_<*C *⇒ ${\hat{G}}_{i}$ = 0 and *S*_*i *_> *C *⇒ ${\hat{G}}_{i}$ = 1. If *S*_*i *_= *C*, we assign the genotype randomly. In the specific example of the Mahalanobis distance, C is usually the solution to

(11) $$\frac{{\left(C-{\hat{\mu }}_{0}^{MLE}\right)}^{2}}{{\hat{\sigma }}_{0}^{2MLE}}=\frac{{\left(C-{\hat{\mu }}_{1}^{MLE}\right)}^{2}}{{\hat{\sigma }}_{1}^{2MLE}}$$

Therefore, by convergence of the MLE, we know that

(12) *lim*_*n *→ ∞ _*C *→_*p *_c*

which will useful for the next section.

### Two Genotype Example: $p_{0}^{*}\neq p_{0}$

We start by assigning genotypes according to their Mahalanobis distance, as done in BRLMM. Recall, we assign subject *i *to genotype 0 if *S*_*i *_<*C*, and to genotype 1 otherwise. Therefore, the probability, $P_{M}^{n}$, that a subject with genotype 1 is *m*isclassified as genotype 0 will be $P_{M}^{n}$ = *P*(*S*_*i *_<*C*|*n*, *θ*, *G*_*i *_= 1), and in the limit, we know $P_{M}^{n}\to \Phi (c*|\mu_{1},\sigma_{1}^{2})\equiv P_{M}^{*}$. Now, it's easy to show that $P_{M}^{*}$ must also be the limiting probability that a subject with genotype 0 is assigned as genotype 1.

(13) $$\begin{array}{c} P(S_{i}>c*|G_{i}=0)=1-\Phi (c*|\mu_{0},\sigma_{0}^{2})\\ =1-\Phi (\frac{c*-\mu_{0}}{\sigma_{0}^{2}}|0,1)=1-\Phi (\frac{\mu_{1}-c*}{\sigma_{1}^{2}}|0,1)\\ =\Phi (\frac{c*-\mu_{1}}{\sigma_{1}^{2}}|0,1)=\Phi (c*|\mu_{1},\sigma_{1}^{2})\equiv P_{M}^{*} \end{array}$$

Therefore, given the genotype, the limiting conditional probabilities, *P*(${\hat{G}}_{i}$ = 1 ⋃ ${\hat{G}}_{i}$ ≠ *G*_*i*_|*G*_*i *_= 0) and *P*(${\hat{G}}_{i}$ = 0 ⋃ ${\hat{G}}_{i}$ ≠ *G*_*i*_|*G*_*i *_= 1) are equal. If *p*_0 _> *p*_1_, then the unconditional probabilities cannot be equal, specifically

(14) $$\begin{array}{c} P({\hat{G}}_{i}=1\cap {\hat{G}}_{i}\neq G_{i})\\ =P({\hat{G}}_{i}=1\cap {\hat{G}}_{i}\neq G_{i}|G_{i}=0)p_{0}\\ >P({\hat{G}}_{i}=0\cap {\hat{G}}_{i}\neq G_{i}|G_{i}=1)p_{1}\\ =P({\hat{G}}_{i}=0\cap {\hat{G}}_{i}\neq G_{i}) \end{array}$$

Obviously the opposite inequality holds if *p*_0 _<*p*_1_. Therefore, with the Mahalanobis distance, the bias will be

(15) $$p_{0}^{*}-p_{0}=P_{M}^{*}p_{1}+(1-P_{M}^{*})p_{0}-p_{0}=P_{M}^{*}(p_{1}-p_{0})$$

**Figure 1:**
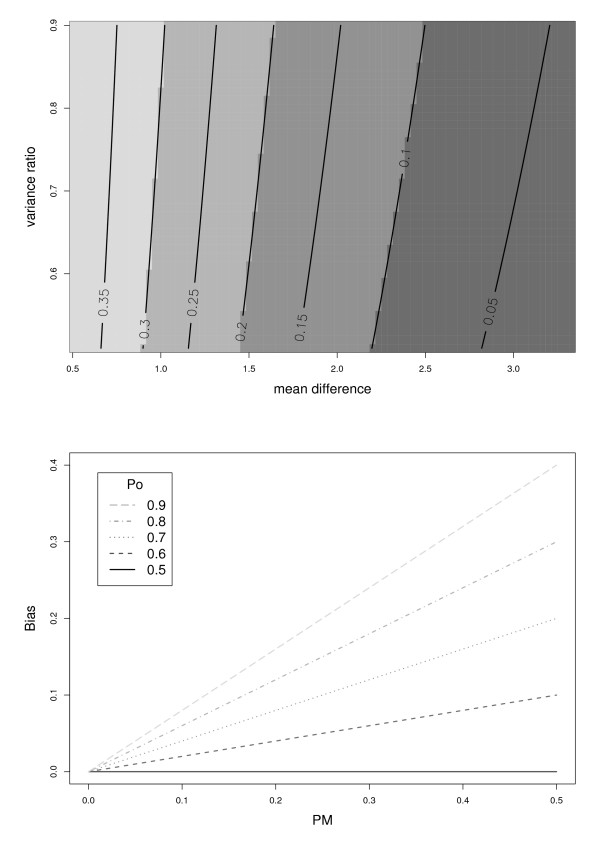
**(*P*_*M *_and Bias) a) *P*_*M *_depends on the ratio, $\sigma_{1}^{2}:\sigma_{0}^{2}$ (y-axis) and on the difference, *μ*_1 _- *μ*_0_(x-axis) **b) The bias, $p_{0}^{*}$ - *p*_0 _(y-axis) depends on *P*_*M *_(x-axis), and is shown for different values of *p*_0_.

Clearly, when *p*_0 _= *p*_1 _= 0.5, $p_{0}^{*}$ for all values of $P_{M}^{*}$. However, when *p*_0 _≠ *p*_1_, the bias is a non-zero function $P_{M}^{*}$, and therefore depends on the parameter group, $\left\{\mu_{1}-\mu_{0},\sigma_{1}^{2}/\sigma_{0}^{2}\right\}$ (Figure 1). As shown by Figure 1, the bias can be quite large when either $P_{M}^{*}$ and/or |p_0 _- 0.5 | is large.

**Figure 2:**
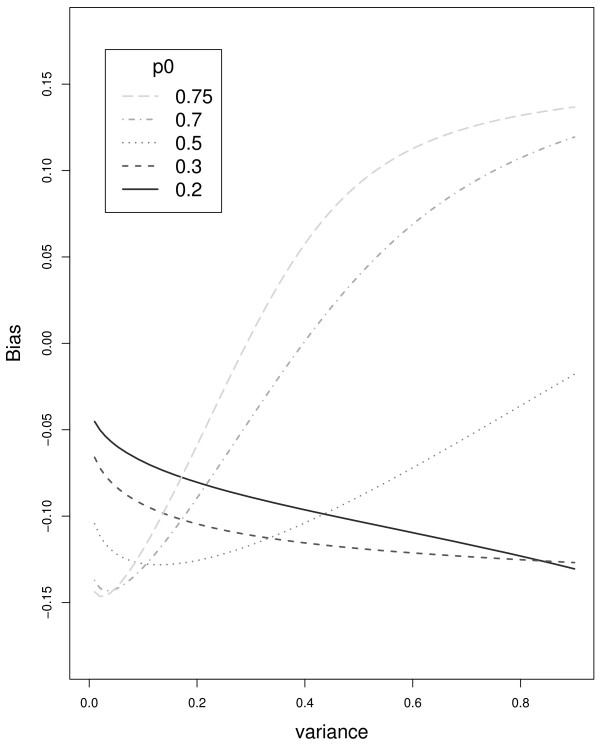
(*P*_0 _and Bias) The bias of the estimate, $p_{0}^{*}=lim_{n\to \infty }\sum_{i=1}^{n}1({\hat{G}}^{i}=0)/n$, depends on *p*_0_, *μ*_1_, *μ*_2_, $\sigma_{0}^{2}$ and $\sigma_{1}^{2}$. After fixing, *μ*_0 _= 0, $\sigma_{0}^{2}$ = 1, and *μ*_1 _= 1, we plot the bias, $p_{0}^{*}$ - *p*_0 _(y-axis), against $\sigma_{1}^{2}$ (x-axis), for different values of *p*_0_.

Next, assume that genotype assignments are based on a likelihood, or weighted likelihood measure. For a value *ω *∈ *c*(0, 1), the probability that *φ*_*g*_(*S*_*i*_) exceeds *ω*, or $2\Phi_{g}(\phi_{g}^{-1}(\omega ))-1$, changes with $\sigma_{g}^{2}$, where $\phi_{g}^{-1}(\omega )$ returns a value greater than *μ*_*g*_. Therefore, *φ*_0_(*s*) = *φ*_1_(*s*) does not imply anything about the relationship between Φ_0_(*s*) = Φ_1_(*s*). We illustrate the potential for bias by a simple example where *μ*_0 _= 0, $\sigma_{0}^{2}$ = 1, and *μ*_1 _= 1. Let *p*_0 _= *p*_1 _= 0.5. Then, for any value of $\sigma_{1}^{2}\neq 1,1-\Phi (\phi_{0}^{-1}(\omega ))\neq \Phi (\phi_{1}^{-1}(\omega ))$. Equivalently, when two normal densities intersect at the threshold value, the probability of misclassifying a genotype 0 subject will not equal the probability of misclassifying a genotype 1 subject, and therefore $lim_{n\to \infty }{\hat{p}}_{0}\neq 0.5=p_{0}$. For a given threshold, c*, we can define the bias by $p_{0}^{*}-p_{0}=p_{0}\Phi_{0}(c*)+p_{1}\Phi_{1}(c*)-p_{0}=p_{0}(\Phi_{0}(c*)-1)+(1-p_{0})\Phi_{1}(c*)$. Unlike the previous example, with the Mahalanobis distance, Figure 2 shows that describing the bias as a function of $\sigma_{2}^{2}$ and *p*_0 _can be difficult. In current tests of association, this inequality shows that it possible for $p_{0}^{n*A}\neq p_{0}^{n*U}$ even when $p_{0}^{A}=p_{0}^{U}$, which as we will see, will lead to an inflated type I error and too many low p-values. Start by noting that GWAS test a surrogate hypothesis, $H_{0}^{*}:p_{0}^{n*A}=p_{0}^{n*U}$, not the true hypothesis of interest, $H_{0}:p_{0}^{A}=p_{0}^{U}$. Because $p_{0}^{n*A}$ need not equal $p_{0}^{n*U}$ when the distributions for cases and controls differ, $H_{0}^{*}$, the tested hypothesis, can be false even when *H*_0 _is true. Let T* be a standard test statistic for $H_{0}^{*}$, which is believed to have the following property, $P(T*>t_{\alpha }^{*}|H_{0}^{*})=\alpha$. Let us make the reasonable assumption that the difference between $P(T*>t_{\alpha }^{*}|H_{0},H_{0}^{*})$ and $P(T*>t_{\alpha }^{*}|H_{0}^{*})$ is small, or, in words, when $H_{0}^{*}$ is known to be true, the validity of *H*_0 _has little effect on the distribution of T*. Then,

(16) $$\begin{array}{ll} P(T*>t_{\alpha }^{*}|H_{0})= & P(T*>t_{\alpha }^{*}|H_{0},H_{0}^{*})P(H_{0}^{*}|H_{0})\\ & +P(T*>t_{\alpha }^{*}|H_{0},H_{1}^{*})P(H_{1}^{*}|H_{0})\\ & \approx \alpha P(H_{0}^{*}|H_{0})+\beta (1-P(H_{0}^{*}|H_{0}))\\ & >\alpha \end{array}$$

Here *β *is a measure of the power to reject $H_{0}^{*}$ and we assume *β *> *α*. Therefore, the current method of rejecting *H*_0 _whenever T* > $t_{\alpha }^{*}$ is actually anti-conservative if the stated p-value is *α*

### Two Genotype Example: Inconsistency

As with any GWAS experiment, we can estimate *p*_0 _and *p*_1 _by

(17) $${\hat{p}}_{0}^{n}\equiv \frac{1}{n}\sum_{i}1({\hat{G}}_{i}=0)\text{ and }{\hat{p}}_{1}^{n}\equiv \frac{1}{n}\sum_{i}1({\hat{G}}_{i}=1)$$

For presentation, we will omit the superscript *n*, writing ${\hat{p}}_{0}^{n}$ and ${\hat{p}}_{1}^{n}$ as ${\hat{p}}_{0}$ and ${\hat{p}}_{1}$. As ${\hat{G}}_{i}$, from equation 5, is equivalent to *E *[*P*(*S*_*i *_<*C*|*n*, *θ*)], we know that

(18) $$lim_{n\to \infty }p_{0}^{n*}\to p_{0}^{*}\text{ and }lim_{n\to \infty }p_{1}^{n*}\to p_{1}^{*}$$

Therefore, by the convergence of $p_{0}^{n*}$ and $p_{1}^{n*}$ to constants and the convergence of the MLE, we have

(19) $$lim_{n\to \infty }{\hat{p}}_{0}\to_{p}p_{0}^{*}\text{ and }lim_{n\to \infty }{\hat{p}}_{1}\to_{p}p_{1}^{*}$$

Having just discussed cases where equation (9) holds, our standard estimates of *p*_0 _and *p*_1 _are not consistent. Specifically,

(20) $$lim_{n\to \infty }{\hat{p}}_{0}{↛}_{p}p_{0}\text{ and }lim_{n\to \infty }{\hat{p}}_{1}{↛}_{p}p_{1}$$

for these scenarios.

### Return of Consistency: Modifying the Mahalanobis Distance

The Mahalanobis Distance, *D*(*g, s*_*i*_|*θ*), measures the conditional probability of getting a value as extreme as *s*_*i *_given genotype g. Therefore, we could achieve the same results using

(21) $$D^{†}(g,s_{i}|\theta )\equiv P(\frac{{\left(S_{i}-\mu_{g}\right)}^{2}}{\sigma_{g}^{2}}>\frac{{\left(S_{i}-\mu_{g}\right)}^{2}}{\sigma_{g}^{2}}|\mu_{g},\sigma_{g}^{2})$$

where *g *∈ {0, 1} and *S*_*i *_is again presumed to be normally distributed. As we saw, the current estimators suffer because they don't account for the genotype probabilities. Borrowing Bayesian terminology, we simply need to weight our distance measure by the prior probability of a subject having each genotype. Therefore, returning to step 4 of our genotyping process, we now define ${\hat{G}}_{i}$ = *g*_*max *_where *g*_*max *_maximizes the function $p_{g}D_{g}^{†}(s)$, a weighted version of the Mahalanobis distance. Let *c*^† ^be the point such that $p_{0}D_{0}^{†}(c^{†})=p_{1}D_{1}^{†}(c^{†})$. Then we are guaranteed consistency as

(22) $$\begin{array}{c} p_{0}^{*}=p_{0}P(S_{i}<c^{†}|G_{i}=0)+p_{1}P(S_{i}<c^{†}|G_{i}=1)\\ =p_{0}(1-0.5D_{0}^{†}(c^{†}))+0.5p_{1}D_{1}^{†}(c^{†})\\ =p_{0} \end{array}$$

With this change, our estimate ${\hat{p}}_{0}\equiv \frac{1}{n}\sum_{i}1({\hat{G}}_{i}=0)$ would now be consistent.

### Dependence/Correlation of Called Genotypes

If we knew {*G*_1_,..., *G*_*n*_}, the true genotypes for a group of *n *subjects at SNP *j*, as opposed to only knowing their called genotypes, $\left\{{\hat{G}}_{1},...,{\hat{G}}_{n}\right\}$, it would easy to construct a test of *H*_0_: $H_{0}:p_{0}^{A}=p_{0}^{U}$ with a specified *α *level. Reject *H*_0 _if $T({\tilde{p}}_{0}^{A},{\tilde{p}}_{0}^{u})>t_{\alpha }$. Here,${\tilde{p}}_{g}^{q}\equiv \frac{1}{n_{q}}\sum_{i:Q_{i}=q}G_{i},t_{\alpha }$ is the 1-*α *percentile of a $\chi_{2}^{2}$ distribution, and

(23) $$T({\tilde{p}}_{0}^{A},{\tilde{p}}_{0}^{U}\equiv {\left(\sqrt{n}\frac{{\tilde{p}}_{0}^{A}}{\sqrt{{\tilde{p}}_{0}^{A}-{\left({\tilde{p}}_{0}^{A}\right)}^{2}}}-\sqrt{n}\frac{{\tilde{p}}_{0}^{U}}{\sqrt{{\tilde{p}}_{0}^{U}-{\left({\tilde{p}}_{0}^{U}\right)}^{2}}}\right)}^{2}$$

The central limit theorem allows us to be confident that we have an *α*-level test because $\sqrt{n}({\tilde{p}}_{g}^{q}-p_{g}^{q})\approx N(0,p_{g}^{q}(1-p_{g}^{q}))$ when {*G*_1_,..., *G*_*n *_is a vector of independent Bernoulli random variables. Again, we have returned to the two genotype scenario to simplify our discussion.

By statements about the perceived *α*-levels of $T({\hat{p}}_{0}^{A},{\hat{p}}_{0}^{U})$, $\left\{{\hat{G}}_{1},...,{\hat{G}}_{n}\right\}$ are often implicitly treated as the true genotypes and are assumed to be a vector of independent Bernoulli random variables. The truth, however, is that ${\hat{G}}_{i_{1}}$ is not independent of ${\hat{G}}_{i_{2}}$. Specifically if C, the threshold for calls, is relatively small, then both *P*(${\hat{G}}_{i_{1}}$ = 1|*C*) and *P*(${\hat{G}}_{i_{2}}$ = 1|*C*) are relatively large. Using this common dependence on C, it is simple to show that ${\hat{G}}_{i_{1}}$ and ${\hat{G}}_{i_{2}}$ are positively correlated.

(24) p(Gˆi1=Gˆi2)=∫C=−∞∞P(Gˆi1j=Gˆi2j|C)f(C)dC=∫C=−∞∞[(p0n*(C))2+(p1n*(C))2]f(C)dC=∫C=−∞∞(p0n*(C))2f(C)dC+∫C=−∞∞(p1n*(C))2f(C)dC≥(∫C=−∞∞(p0n*(C))f(C)dC)2+(∫C=−∞∞(p1n*(C))f(C)dC)2=(p0n*)2+(p1n*)2

This proof, which uses Jensen's inequality, clearly shows that the two variables, ${\hat{G}}_{i_{1}}$ and ${\hat{G}}_{i_{2}}$, are not independent as that would have implied

$P({\hat{G}}_{i_{1}}={\hat{G}}_{i_{2}})=P({\hat{G}}_{i_{1}}=0,{\hat{G}}_{i_{2}}=0)+P({\hat{G}}_{i_{1}}=1,{\hat{G}}_{i_{2}}=1)={\left(p_{0}^{n*}\right)}^{2}+{\left(p_{1}^{n*}\right)}^{2}$. The consequence of this dependence is that $\sqrt{n}({\hat{p}}_{g}^{q}-p_{g}^{q})$ does not follow a $N(0,p_{g}^{q}(1-p_{g}^{q}))$ distribution, and in turn, that $T({\hat{p}}_{0}^{A},{\hat{p}}_{0}^{U})$ neither follows a $\chi_{2}^{2}$ distribution nor has *P*($T({\hat{p}}_{0}^{A},{\hat{p}}_{0}^{U})$ > *t*_*α*_) = *α*.

We next examine the behavior of ${\hat{p}}_{0}^{A},{\hat{p}}_{0}^{U}$, and $T({\hat{p}}_{0}^{A},{\hat{p}}_{0}^{U})$. First, as is common, the dependence increases the variance of these estimates. For any population,

(25) $$\begin{array}{ll} var(\sqrt{n}\frac{\sum_{i=1}^{n}1({\hat{G}}_{i}=0)}{n}) & \\ & =nE[var(\frac{\sum_{i=1}^{n}1({\hat{G}}_{i}=0)}{n}|C)]\\ & +nvar(E[\frac{\sum_{i=1}^{n}1({\hat{G}}_{i}=0)}{n}|C]) \end{array}$$

The first term, $nE[var(\frac{\sum_{i=1}^{n}1({\hat{G}}_{i}=0)}{n}|C)]$, represents the uncertainty in the true number of subjects with genotype 0, and can be roughly approximated by ${\hat{p}}_{0}-{\left({\hat{p}}_{0}\right)}^{2}$. The second term, $nvar(E[\frac{\sum_{i=1}^{n}1({\hat{G}}_{i}=0)}{n}|C])$ reflects that there will be a subpopulation, of a random size ~*n*|Φ^*M*^(*C*) - Φ^*M *^(*E*[*C*])|, that is assigned the 'non-ideal' genotype, where a call is labeled 'non-ideal' if it would have been different had the threshold been *E *[*C*]. We can approximate this second term, the overall increase in the variance, by *nvar*(Φ^*M*^(*C*)) if *P*(${\hat{G}}_{i}$ = 0|*C*) ≈ Φ^*M*^(*C*) and the *cor*(${\hat{G}}_{i_{1}}$, ${\hat{G}}_{i_{2}}$|*C*) ≈ 0. In experiments, we can estimate *nvar*(Φ^*M*^(*C*)) by bootstrapping samples of C or ${\hat{\Phi }}^{M}$(*C*).

To focus on the distributions instead of just the variances, we decompose $\sqrt{n}({\hat{p}}_{0}-p_{0}^{n*})$ as

(26) $$\begin{array}{l} \sqrt{n}({\hat{p}}_{0}-p_{0}^{n*})=\\ \sqrt{n}({\hat{\Phi }}^{M}(C)-E[{\hat{\Phi }}^{M}(C)])=\\ \sqrt{n}({\hat{\Phi }}^{M}(C)-\Phi^{M}(C))+\Phi^{M}(C)-\Phi^{M}(E[C]))+\\ \left(\Phi^{M}(E[C])-E[{\hat{\Phi }}^{M}(C)])\right) \end{array}$$

Note that ${\hat{p}}_{0}\equiv {\hat{\Phi }}^{M}(C)$ and $p_{0}^{n*}\equiv E[{\hat{\Phi }}^{M}(C)]$. The appropriate multiple of the first term, *n*(${\hat{\Phi }}^{M}$(*C*) - Φ^*M*^(*C*)), should be well approximated by *X*-E [C], where *X *~binomial(*n*, *E [C]*). From our own experience, we have seen that the third term, (Φ^*M*^(*E*[*C*]) - *E*${\hat{\Phi }}^{M}$(*C*)]), is a constant close to 0. The second term, (Φ^*M*^(*C*) - Φ^*M*^(*E*[*C*])) is the variable which causes deviation from normality. Again, we could approximate the distribution of this term by bootstrapping *C*.

Next, we offer an example to demonstrate the effect of dependence among called genotypes. Specifically, using the IBD data, we show that a $N(0,E[{\hat{p}}_{AB}](1-E[{\hat{p}}_{AB}]))$ is a poor approximation for the distribution of $\sqrt{n}({\hat{p}}_{AB}-E[{\hat{p}}_{AB}])$. Because we will use real data, we have purposely chosen to discuss $\sqrt{n}({\hat{p}}_{AB}-E[{\hat{p}}_{AB}])/\sqrt{E[{\hat{p}}_{AB}]-{\left(E[{\hat{p}}_{AB}]\right)}^{2}}$ instead of *T *. There are many reasons that *T *may not follow a $\chi_{2}^{2}$ distribution, including $p_{g}^{*}$ being a poor estimate of *p*_*g*_, the distributions of *S*_*i *_being far from normal and population substructure. However, for large *n*, the only reason that $\sqrt{n}({\hat{p}}_{AB}-E[{\hat{p}}_{AB}])/\sqrt{E[{\hat{p}}_{AB}]-{\left(E[{\hat{p}}_{AB}]\right)}^{2}}$ will not be approximately normal is if $\left\{{\hat{G}}_{1},...,{\hat{G}}_{n}\right\}$ are not independent. Also, we chose to focus on the AB genotype as this is certain to be one of the genotypes with an overlapping cluster.

**Figure 3:**
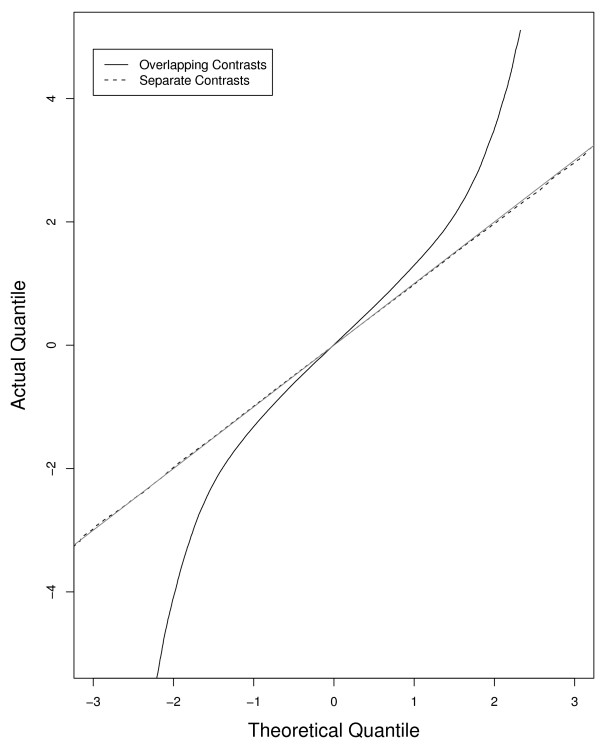
**(Dependency of Calls) The density of $\sqrt{n}({\hat{p}}_{AB}-E[{\hat{p}}_{AB}])/(E{\hat{p}}_{AB}-E[{\hat{p}}_{AB}^{2}])$ is compared to a N(0,1) density for 500 subject samples in a quantile-quantile plot**. The deviation from the Y = X line indicates that $n{\hat{p}}_{AB}$ is not distributed as a binomial(n, *p_*AB*_*) variable.

For each of our 3137 SNPs in the IBD data, we bootstrap 40 samples of 500 subjects, and calculate 40 values of $\sqrt{n}({\hat{p}}_{AB}-E[{\hat{p}}_{AB}])/\sqrt{E[{\hat{p}}_{AB}]-{\left(E[{\hat{p}}_{AB}]\right)}^{2}}$, where $\text{E}[{\hat{p}}_{AB}]$ is estimated by $\frac{1}{40}\sum_{k=1}^{40}{\hat{p}}_{AB}$. The qq-plot in Figure 3 compares these 40 × 3137 values with a N(0,1). The distribution is far from normal, which implies that $\left\{{\hat{G}}_{1},...,{\hat{G}}_{n}\right\}$ are dependent. Some SNPs were more likely to contribute skewed values than others, but the top and bottom 100 values (200 total) are from 64 different SNPs, indicating that no one SNP, or small number of SNPs, is responsible for the deviation in the qq plot. In contrast, the qq-plot from well-behaved SNPs, where the contributions to the density of S from the three genotypes were separated, was the expected straight line, showing that it was not the normal approximation skewing the results (Figure 3). Because the magnitude of the observed values were larger than predicted by theory, the practical implication is that tests based on the statistic, *T*, estimated by $F_{\chi_{2}^{2}}$(*T*), will be anti-conservative(i.e. too many significant p-values after adjusting for multiple testing) under the null hypothesis. Here we also note that if *S*_*i *_were truly normal, the impact of the dependency would be much less. For more details on the origin of dependency, please see Table 1.

### Comparing Tests of Association

Table 2 shows the results from the simulations, and lists the percentage of the influential SNPs that were ranked among the top 100 most significant SNPs, where the rankings were determined by either LogiCALL, a likelihood ratio test, or a standard test, similar to Bead-Studio. As there were 100 influential SNPs in each simulation, an ideal scenario would have 100% of the influential SNPs in the top 100 SNPs. As expected, the percentages increase as the relative risk, comparing the two homogeneous genotypes, increases from 1.5 to 2.5. So long as the densities of *φ *(M_*ji*_|*G*_*ji *_= *AA*) and *φ *(*M*_*ji*_|*G*_*ji *_= *AB*) were distinct, which was the case when the distance between *μ*_*AA *_and $(\mu_{AB}^{A}+\mu_{AB}^{U})/2$ exceeded 0.5 (and was less than 1.239), all three tests performed equally well. As the shifts were decreased in simulations, many of these shifted SNPs started to appear in the top 100 most significant SNPs, when ranked by a standard test. Furthermore, the loss in power was exaggerated when the amount of overlap differed between cases and controls, *φ*(*M*_*ji*_|*G*_*ji *_= *AB*, *Q*_*i *_= *A*) ≠ *φ *(*M*_*ij*_|*G*_*ij *_= *AB*, *Q*_*i *_= *U*). In the most extreme case, when *E *[*M*_*ji*_|*G*_*ji *_= *AB*] = -0.539 and *E *[*M*_*ji*_|*G*_*ji *_= *AB*, *Q*_*i *_= *A*] - *E *[*M*_*ji*_|*G*_*ji *_= *AB*, *Q*_*i *_= *U*] = 0.2, the standard test only detected about half as many influential genes as it did when there were no shifted genes. In contrast, LogiCALL almost never ranked any of the shifted SNPs in the top 100. However, had these shifted SNPs been influential, LogiCALL would have had less power to detect them. The performance of the likelihood ratio was in between the two other tests, but performed nearly as well as LogiCALL when *φ *(*M*_*ji*_|*G*_*ji *_= *AB*, *Q*_*i *_= *A*) = *φ *(*M*_*ij*_|*G*_*ij *_= *AB*, *Q*_*i *_= *U*).

**Table 2:** The percentage of influential genes among the top 100 most significant SNPs, as ranked by LogiCALL, a likelihood ratio test, and a standard test.

		RR = 1.5			RR = 2.0			RR = 2.5		
Shift	Difference	LogiCALL	LR	Standard	LogiCALL	LR	Standard	LogiCALL	LR	Standard
0.5	0	4.4	4.6	4.5	41.8	41.8	41.3	78.8	79.2	79.3
	0.2	4.4	4.6	4.5	42.0	41.8	41.3	78.8	79.3	79.4
										
0.3	0	4.4	4.6	4.6	42.0	41.9	41.2	78.8	79.2	79.4
	0.2	4.4	1.9	0.2	41.6	29.5	14.4	78.8	67.3	42.7
										
0.2	0	4.4	4.4	2.1	42.0	41.4	25.9	78.8	78.9	47.1
	0.2	3.7	0.1	0.0	38.9	3.4	0.0	75.3	21.3	0.0

Simulated data sets are full described in the *Methods Section*. The shift is the distance between the and *μ*_*AA *_and $(\mu_{AB}^{A}+\mu_{AB}^{U})/2$. The Difference is the distance between $\mu_{AB}^{A}$ and $\mu_{AB}^{U}$. RR is the genotype relative risk for subjects homogeneous for the minor allele.

In GWAS, each marker is tested for association with the disease. Here, we compare four methods for testing the 3137 chosen SNPs in the IBD study. In the first method, we let Bead-Studio call the genotypes and perform its standard Cochran-Armitage test. Default settings were used to assign calls as missing and remove poor quality SNPs. In the second method, we call the SNPs using logiCALL and test for association using logistic regression, although using Cochran-Armitage would not change our conclusions. In the third method, we calculate the Likelihood Ratio Statistic comparing ${\hat{\theta }}^{j}$ and ${\hat{\theta }}_{R}^{j}$ from equation (3) using all SNPs, and in the fourth method, we calculate the LR statistic using only those SNPs where ${\hat{\theta }}^{j}$ and ${\hat{\theta }}_{R}^{j}$ yield identical calls, and Hardy-Weinberg equilibrium, for controls alone, is not violated at a statistical significance level of < 10^-16^. For each method, we calculated the proportion of 'p-values' that were less than 0.005, 0.001, and 0.0005 (Table 3).

**Table 3:** The percentage of 'p-values' less than traditional^ α^-levels (0.005,0.001,0.005) are listed for four tests of association.

Method	n	p < 0.005	p < 0.001	p < 0.0005
BeadStudio	3487	0.015	0.005	0.004
logiCALL	5533	0.004	0.002	0.002
LR	5533	0.087	0.061	0.052
LR-(same)	3014	0.006	0.002	0.001

1) BeadStudio (default setting for missing assignements and omitting SNPs).

2) logiCALL.

3) Likelihood Ratio (LR) using all SNP.

4) Likelihood ratio (LR-same) using only SNP where calls were the same for restricted and unrestricted parameter sets.

When genotyping all SNPs and all subjects, the proportion of Bead-Studio 'p-values' below the three thresholds far exceeded 0.005, 0.001, and 0.0005. Even after removing low quality SNPs and allowing missing calls, the proportion of 'p-values' below the three thresholds were 0.015, 0.005, and 0.004. In contrast, LogiCALL eliminated nearly all false positives. The proportion below the three thresholds were 0.004, 0.002, and 0.002. If the majority of these SNPs are presumed to be null associations, logiCALL appears to be the superior method, so long as the power loss is minimal. Assigning conservative 'p-values' to problematic SNPs is nearly equivalent to removing them. However, because of the tendency for there to be multiple, nearly equivalent, maximum likelihood estimates, the relative distances to the AA, AB, and BB genotypes using the single set of maximum likelihood estimates may not be adequate in identifying questionable calls. Therefore, logiCALL gains an advantage by combining two methods for identifying suspect calls. Additionally, it avoids false positives caused by differential bias, where the proportion of missing calls differs between cases and controls. This new method simplifies testing by requiring no preprocessing and testing all SNPs. The power loss from logiCALL depends on the quality of the data. When the statistic for a SNP cleanly separates into an AA, AB, and BB group, there is no power loss. In our IBD example, the 'p-values' reported for rs2066843 and rs2076756, the two SNPs that are believed to be truly associated with IBD were similar for the two methods, Bead-Studio (2.9 × 10^-9 ^and 5.1 × 10^-10^) and logiCALL (1.5 × 10^-8 ^and 1.6 × 10^-9^). Among those subjects meeting the 96% Bead-Studio call rate, logiCALL found no questionable calls.

The likelihood ratio method had mixed results. Clearly, when the distribution of the summary statistic is a mixture of normals, the estimated genotype proportions are asymptotically unbiased. Unfortunately, this method still resulted in an increased number of false positives. However, if we removed those SNPs where at least one call changed when switching from $\hat{\theta }$ to ${\hat{\theta }}_{R}$, the false positive rate decreased to the expected level. In theory, all calls should be identical and only the resulting likelihoods should differ. When assigning genotypes, the cost, in likelihood, incurred from forcing the vectors of genotype proportions to be equal should be far less than the cost of switching calls. When the reverse is true, and calls switch, the statistic for the three groups cannot be well separated, and the p-value is suspect.

## Conclusion

In genome-wide association tests, under the null hypothesis, the test statistic rarely follows the expected chi-squared distribution. This deviation tends to result in an excess of false positives. Unfortunately, the investigation into the origin of this deviation has yet to be completed. The problems associated with poor signal quality and population substructure have been thoroughly explored. However, the overlap of fluorescent signals has only been identified as a serious problem, and has yet to be fully explained. In this paper, we have provided two reasons, parameter inconsistency and called genotype dependency, that help explain how overlap causes this deviant behavior. Furthermore, we propose two methods, logiCALL and a method based on the likelihood ratio statistic that better handle the problems of inconsistency and dependency. These methods will perform similarly to the common, genotype-based, test statistics for the well-behaved SNPs and appear to create fewer false positives for the difficult-to-call SNPs. We have also identified a new characteristic of some false positives, that the call differs when using ${\hat{\theta }}_{R}^{j}$ vs. ${\hat{\theta }}^{j}$, that will help distinguish which low p-values represent significant disease/marker association.

We have demonstrated that increasing sample size alone will not eliminate type I error, as genotyping, in its current form, leads to inconsistent estimates of population parameters. To alleviate this inconsistency, the distance measure used for assignment would need to be switched to $p_{g}d_{g}^{†}(s)$, defined in the *Results and Discussion Section*. Moreover, we have proven that the called genotypes can be dependent under certain conditions, and that tests based on called genotypes need to account for the increased variance caused by dependence. Finally, we illustrated that the likelihood profile of the data can be relatively flat near the MLEs. Therefore, judging the quality of calls from distances based only on the MLE may not provide an adequate means to identify questionable calls. Hence logiCALL, which looks at all locally-maximal likelihood estimates of the parameters can reduce the type I error rate. Testing association by the likelihood ratio statistic is another promising method for addressing the problems associated with overlapping signals.

## Availability and requirements

Computer programs are available on author's website, .

## Authors' contributions

JS identified the original problem. Both JS and HZ developed the problem. JS drafted the manuscript. Both authors read and approved the final manuscript.
